# Prediction of outcomes for rescue cerclage in cervical insufficiency: A multicenter retrospective study

**DOI:** 10.7150/ijms.87941

**Published:** 2024-03-25

**Authors:** Subeen Hong, Hyun Sik Chung, Sujin Choi, Yun Sung Jo

**Affiliations:** 1 Department of Obstetrics and Gynecology, Seoul St. Mary's Hospital, College of Medicine, The Catholic University of Korea, 06591 Seoul, Republic of Korea.; 2 Department of Anesthesiology and Pain Medicine, Eunpyeong St. Mary's Hospital College of Medicine, The Catholic University of Korea, 06591 Seoul, Republic of Korea.; 3 Department of Obstetrics and Gynecology, St. Vincent's Hospital, College of Medicine, The Catholic University of Korea, 06591 Seoul, Republic of Korea.

**Keywords:** cervical insufficiency, cerclage, rescue cerclage, predictive model, preterm birth, outcome, prognosis

## Abstract

**Purpose:** Cervical insufficiency is a significant risk factor for preterm birth and miscarriage during the second trimester; cervical cerclage is a treatment option. This study seeks to evaluate the predictive roles of various clinical factors and to develop predictive models for immediate and long-term outcomes after rescue cerclage.

**Methods:** We conducted a multicenter retrospective study on patients who underwent rescue cerclage at 14 to 26 weeks of gestation. Data were collected from the Electronic Medical Record systems of participating hospitals. Outcomes were dichotomized into immediate failure (inability to maintain pregnancy for at least 48 hours post-cerclage, gestational latency < 2 days) and long-term success (maintenance of pregnancy until at least 28 weeks of gestation). Clinical factors influencing these outcomes were analyzed.

**Results:** The study included 98 patients. Immediate failure correlated with longer prolapsed membrane lengths, elevated C-reactive protein levels at admission, and extended operation time. The successful maintenance of pregnancy until at least 28 weeks was associated with earlier gestational age at diagnosis, negative AmniSure test results, longer lengths of the functional cervix, and smaller cervical dilatation at the time of cerclage. Binary logistic regression models for immediate failure and long-term success exhibited excellent and good predictive abilities, respectively (AUROC = 0.912, 95% CI: 0.834-0.989; and AUROC = 0.872, 95% CI: 0.788-0.956).

**Conclusion:** The developed logistic regression models offer a valuable tool for the prognostic assessment of patients undergoing rescue cerclage, enabling informed clinical decision-making.

## Introduction

Preterm birth is a critical obstetric issue accounting for about 7% of total births 1, with approximately 1% caused by cervical insufficiency 2. Patients with cervical insufficiency exhibit weak cervical tissue integrity, resulting in painless cervical dilation during the mid-trimester. This condition can lead to premature expulsion of the conceptus from the uterine cervix, culminating in either miscarriage or early preterm birth. Cervical cerclage is a surgical treatment for cervical insufficiency which was first introduced by Shirodkar and McDonald 3, 4. If cervical cerclage is applied to a woman whose cervix is already dilated, such a treatment is referred to as physical examination-indicated cerclage, also known as rescue cerclage. Comparative studies of the Shirodkar and McDonald cerclage methods have revealed no significant disparities in perinatal outcomes between the two techniques 5. Furthermore, the choice of cerclage stitch material—whether monofilament or braided suture—appears to have no impact on the rate of preterm births 6. Despite previous uncertainties surrounding the advantages of rescue cerclage, contemporary research—including a randomized controlled trial 7 and a meta-analysis 8—has substantiated its efficacy in prolonging gestation and enhancing neonatal outcomes compared to expectant management. Nevertheless, the procedure is challenging and predisposes patients to complications such as membrane rupture and cervical laceration. The likelihood of preterm birth remains high post-surgery, with a mean gestational age at delivery of 30.6 weeks. Furthermore, the incidence of extremely preterm delivery, defined as less than 24 weeks of gestation, stands at 23%—comparable to rates observed with expectant management 8. The prognostic factors influencing the success rate of rescue cerclage remain underexplored 9-12. Hitherto, only a handful of studies have forecasted the outcomes following rescue cerclage 13, 14.

Our study examines the prognostic significance of various clinical factors in patients who have undergone rescue cerclage and aims to propose a predictive model for both the immediate and long-term outcomes post-cerclage.

## Materials and Methods

### Study design and participants

This multicenter retrospective study was conducted among patients who underwent rescue cerclage during 14 to 26 weeks of gestation at Seoul St. Mary's Hospital and St. Vincent's Hospital, two hospitals under the College of Medicine, Catholic University of Korea, from January 2009 to March 2021. The following inclusion criteria were applied: singleton gestation, live fetus, and intact membrane. The following exclusion criteria were applied: with major fetal congenital anomaly, clinically suspected chorioamnionitis, and regular painful contractions at the time of diagnosis of cervical insufficiency. This study was approved by the Institutional Review Board (approval number: XC21RID10161).

### Data collection and definition of clinical diagnosis

Data was obtained from the Electric Medical Record (EMR) of the hospitals, which included information about patient demographic characteristics, laboratory and radiology test results (e.g., ultrasound findings).

Clinically suspected chorioamnionitis was defined as a disorder with fever, uterine fundal tenderness, maternal tachycardia (>100 beats/min), fetal tachycardia (>160 beats/min) and purulent or foul amniotic fluid 15. The results of AmniSure test (PAMG-1 immunoassay, AmniSure ROM test, N-Dia Inc., NY, USA) before cerclage were collected. Vaginal ultrasound was used to measure the width (A) and length (B) of prolapsed membrane and the length (C) of the functional cervix that was open with its shape maintained. After rescue cerclage, vaginal ultrasound was used to measure cerclage height, defined as the distance between the external cervix and a stitch of cerclage. We analyzed immediate outcome, defined as the failure to maintain pregnancy for at least 48 hours post-cerclage (gestational latency < 2 days), and long-term outcome, defined as the success in sustaining pregnancy until at least 28 weeks of gestation.

### Statistical analysis

Data was presented as means ± standard deviations or absolute values (proportions), as appropriate after using Shaprio-Wilks test to assess normality. To compare two groups, Student's t-test was used for continuous variables, while Chi-squared test was used for categorical variables. Binary logistic regression was done to calculate predictive models for the immediate and long-term outcomes based on results which demonstrated a* p*-value of <0.1 on univariate analyses. The diagnostic accuracy and the optimal cutoff values of the failure and success predictive models were analyzed using area under the receiver operating characteristic (AUROC). To evaluate fitness of the models, Hosmer and Lemeshow goodness of fit (GOF) test and values of R square of models were used. All *p*-values were two-sided, and* p <* 0.05 was considered statistically significant. Statistical analyses were performed using R software version 4.0.2 (R Foundation for Statistical Computing, Vienna, Austria).

## Results

### General characteristics

**Figure 1:**
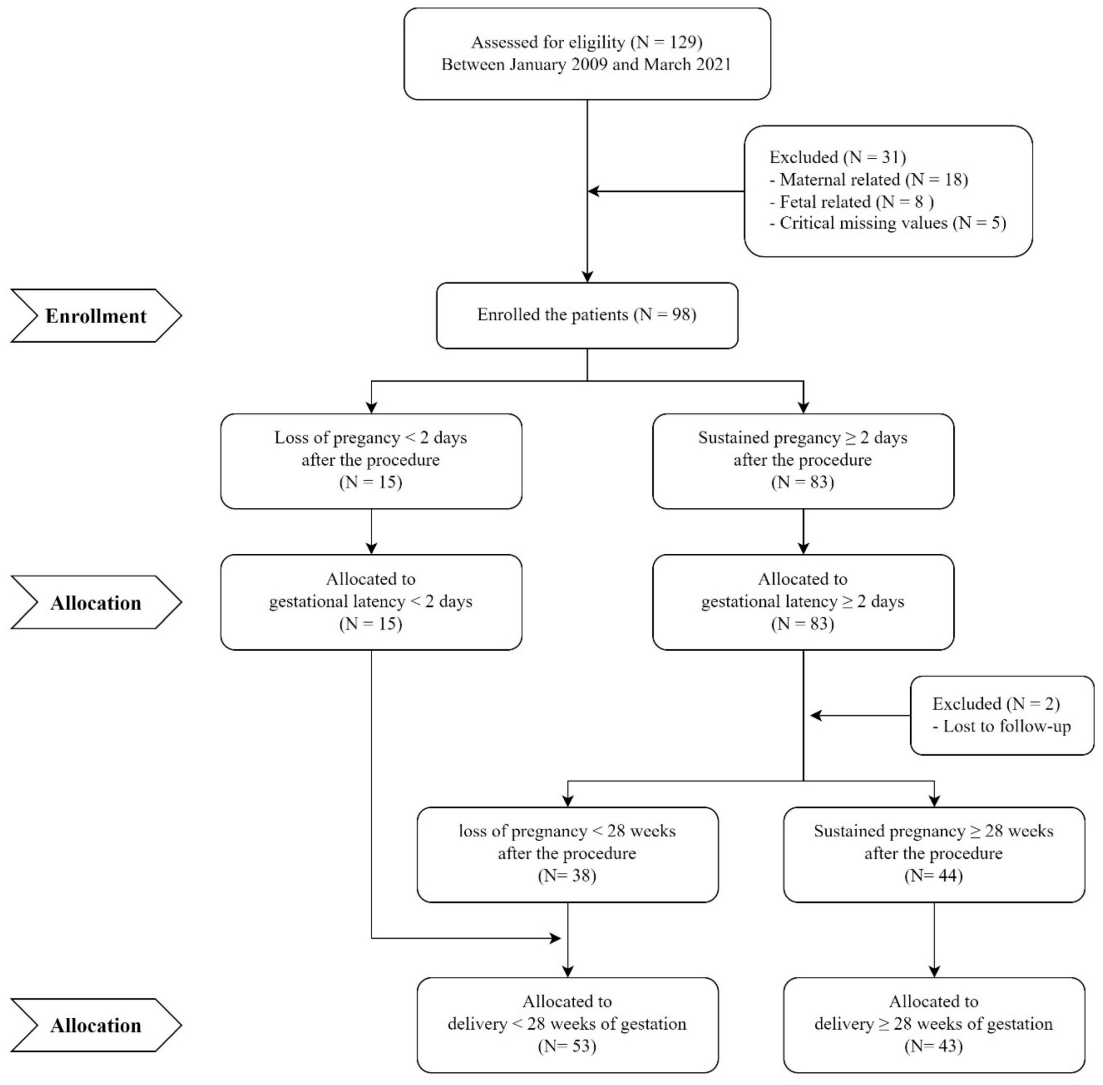
CONSORT flow diagram of participant in this study.

A total of 129 patients that fulfilled the incision criteria were included in the analysis. The procedures were performed by a total of 5 maternal fetal medicine specialists, who had 8 to 25 years of clinical experience at the time of the procedure. The McDoanld's operation was performed in all cases. Among these patients, 31 were excluded, 98 patients were included in the immediate outcome analysis, and 96 patients were included in the long-term outcome analysis after 2 patients were lost to follow-up (Figure 1). General characteristics of the patients are shown in Table 1. The mean gestational age at diagnosis was 21.8 ± 2.6 weeks, while the mean gestational age at delivery was 28.3 ± 6.9 weeks. There were 52 (53.1%) nullipara patients. Upon physical examination, the mean cervical dilation size was 2.8 ± 1.2 cm, and majority (89.8%) had a prolapsed membrane. The mean width (A) and length (B) of prolapsed membrane measured by ultrasound was 2.2 ± 1.8 cm and 1.3 ± 1.4 cm, respectively. The mean operating time taken for rescue cerclage was 27.7 ± 18.6 min. After rescue cerclage, the cervical length was 2.1 ± 0.9 cm, and the cerclage height was 1.3 ± 0.5 cm.

### Predictors and a predictive model for the immediate outcome

Fifteen patients (15.3%) experienced the failure to maintain pregnancy until 48 hours after cerclage, defined as gestational latency of less than 2 days. Two patients had failure due to intraoperative membrane rupture. If the gestational latency is more than 2 days, there is a significantly higher gestational age at diagnosis (22.0 ± 2.5 vs. 20.5 ± 3.1 weeks, *p*-value = 0.023), fewer symptoms at diagnosis (p-value = 0.008), and lower positive rates of AmniSure (*p*-value = 0.037) compared to failure patients (Table 1). Moreover, there is a display of a smaller size of cervical dilation (2.7 ± 1.1 vs. 3.4 ± 1.1 cm, *p*-value = 0.049) on physical examination, along with smaller sizes of A (1.9 ± 1.6 cm vs. 3.9 ± 2.0 cm, *p*-value < 0.001) and B (1.0 ± 1.0 vs. 2.9 ± 2.0 cm, *p*-value = 0.003) of the prolapsed membrane on ultrasound. Additionally, there is a significantly shorter operating time (25.6 ± 17.2 vs. 40.0 ± 22.1 min, *p*-value = 0.005). After rescue cerclage, cervical length, and cerclage height show no significant differences between the group with gestational latency less than 2 days and the group with gestational latency of 2 days or more. No differences are observed in anesthetic technique (general vs. regional anesthesia; *p*-value = 0.143), by the performed operator (*p*-value = 0.200), and the time taken between the operation started and the diagnosis for cervical insufficiency between both groups (20.8 ± 28.5 vs. 23.5 ± 47.4 h, respectively, *p*-value = 0.768).

**Figure 2:**
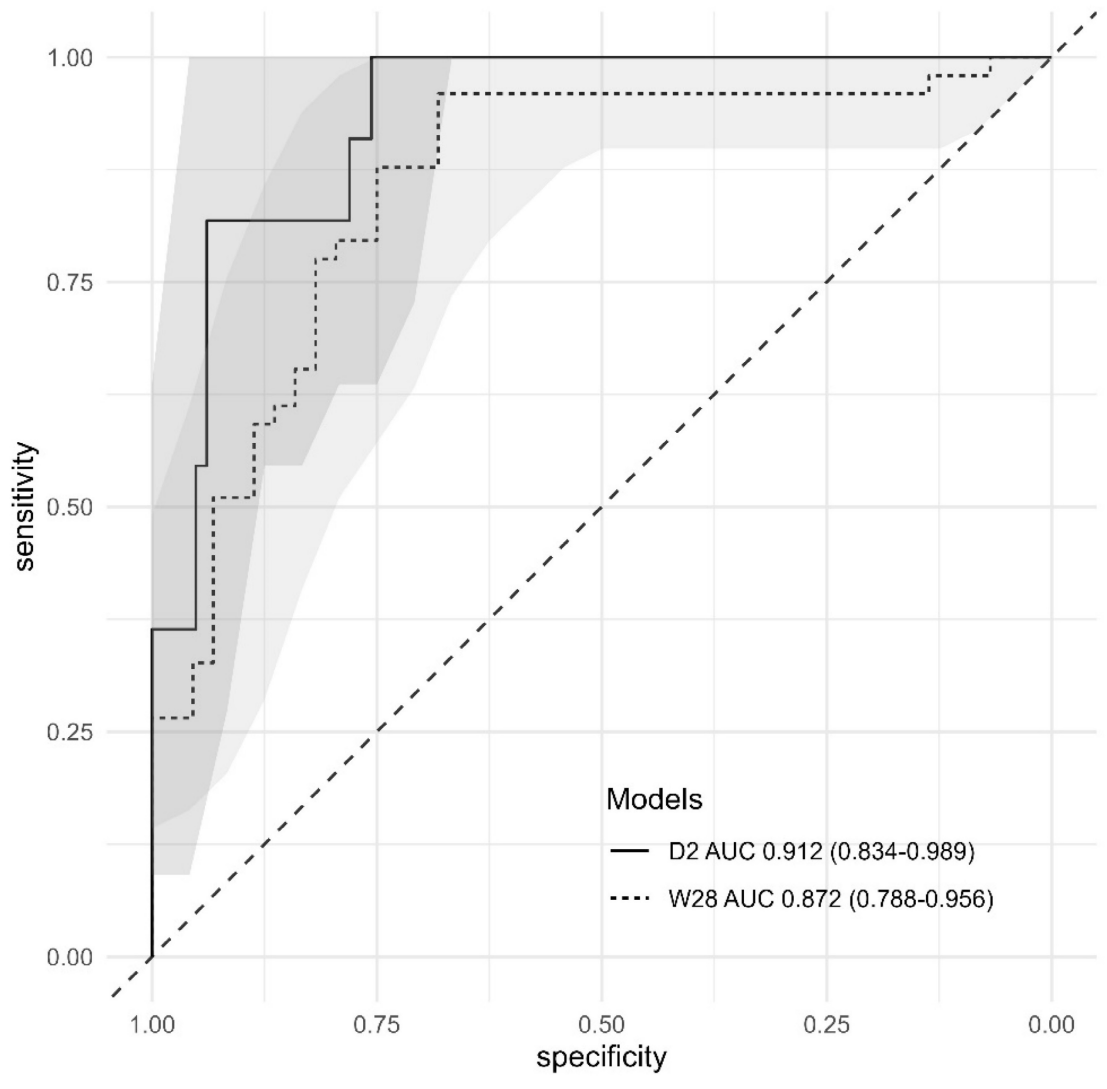
Receiver operating characteristic curve of the predictive models of immediate failure (gestational latency < 2 days, D2) and long-term success (W28) after rescue cerclage. The light color areas indicate 95 % confidence regions for the ROCs.

**Figure 3:**
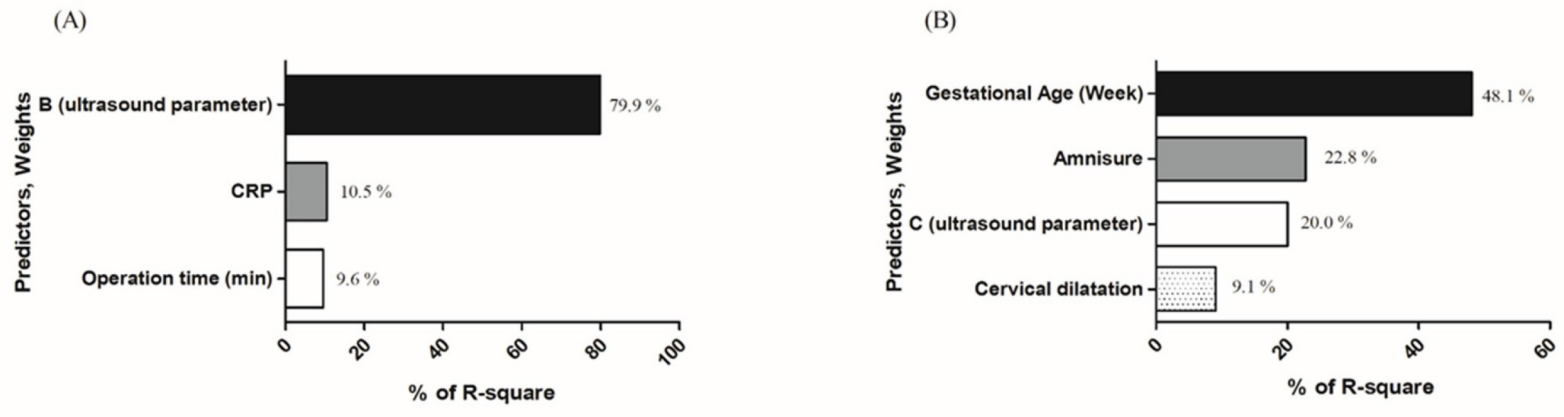
Predictors for of the predictive models after rescue cerclage from results of binary logistic regression. (A). Individual weight of predictors in the immediate failure model. (B) Individual weight of predictors in the long-term success model.

A predictive model for immediate failure, defined as gestational latency of less than 2 days, was calculated using binary logistic regression through stepwise and backward approaches, incorporating parameters that showed statistical significance on univariate analysis (Table 1, Table 3). The predictive accuracy and the most discriminatory cutoff values to predict the failure were determined using AUROC. The predictive accuracy of model was 0.912 (95% CI: 0.834-0.989) (Figure 2). In the model, the most discriminatory cutoff values of CRP at admission, length (B) of the “bulging amniotic sac,” and operation time were 4.3 mg/dL, 2.9 cm, and 20 min, respectively (Table 4). With these cutoff values, the model showed a sensitivity of 70.6%, specificity of 100%, positive predictive value (PPV) of 31.0%, and negative predictive value (NPV) of 100% (Table 4). For model fitness, the value of R square was 0.3778430, while the ꭓ^2^ and *p*-values of GOF test for the model were 2.949 and 0.938, respectively. Figure 3 showed the individual weight of predictors in the model according to the value of R square (Figure 3-A).

### Predictors and predictive model for the long-term outcome

We analyzed 96 patients, excluding 2 who did not have delivery information (Figure 1).

In cases where gestational latency exceeded 8 weeks, there were 37 patients. They were found to be younger in age compared to those with gestational latency of less than 8 weeks (33.5 ± 4.7 vs. 31.9 ± 2.7, *p*-value=0.049). In cases where symptoms were present at the time of diagnosis, the incidence was lower (86.9% vs. 45.9%, *p*-value=0.008). Additionally, patients in this group exhibited lower white blood cell (WBC) counts (11.175 ± 3.207 vs. 0.987 ± 0.248, *p*-value=0.045), lower C-reactive protein (CRP) levels (1.13 ± 1.59 vs. 0.56 ± 0.53, p-value=0.021), and smaller measurements for both A and B in membrane bulging (A: 2.74 ± 1.87 vs. 1.44 ± 1.62, *p*-value=0.003; B: 1.80 ± 1.65 vs. 0.67 ± 0.99, *p*-value=0.001). Furthermore, post-cerclage, cervical height was longer (1.25 ± 0.46 vs. 1.57 ± 0.42, *p*-value=0.005) (Table 2).

Cases of long-term success, defined as delivery at a gestational age of 28 weeks or more, accounted for 43 (44.8%). The success group had a higher gestational age at diagnosis (22.0 ± 2.2 vs. 21.0 ± 2.6 weeks, p-value < 0.001), fewer symptoms at diagnosis (32.6% vs. 64.2%, p-value = 0.004), lower WBC counts, lower CRP levels (Table 2), and fewer cases of AmniSure positivity (11.6% vs. 32.0%, p-value = 0.002) than those with a gestational age of less than 28 weeks. The success group also had a smaller size of cervical dilatation (2.5 ± 1.1 vs. 3.1 ± 1.2 cm, *p*-value = 0.041), smaller sizes (A and B) of prolapsed membrane (Table 2), shorter operation time (22.0 ± 15.5 vs. 31.7 ± 20.3 min, *p*-value = 0.012), and higher cerclage height after surgery (1.5 ± 0.4 vs. 1.1 ± 0.2 cm, *p*-value = 0.005). The operator (*p*-value = 0.309), anesthetic technique (*p*-value = 0.270), and the time taken until operation after the diagnosis of cervical insufficiency (19.5 ± 34.0 vs. 24.5 ± 53.6 h, respectively, *p*-value = 0.586) were not different between the two groups.

Binary logistic regression was performed using the univariate analysis results by a stepwise and backward approach. The predictive model for success was calculated using binary logistic regression results (Table 3). The predictive accuracy and most discriminatory cutoff values to predict the success of cerclage were determined using AUROC. The predictive accuracy of the model for success was 0.872 (95% CI: 0.719-0.984) (Figure 2). The most discriminatory cutoff values for each variable were as follows: 21 weeks and 4 days of gestational age, negative AmniSure test, C of 4 cm on ultrasound, and 3 cm cervical dilatation. With these cutoff points, the model showed a sensitivity of 88.4%, specificity of 73.3%, PPV of 82.6%, and NPV of 81.5%, respectively (Table 4). For model fitness, the value of R square of the model was 0.29, whereas the ꭓ^2^ and *p*-value of GOF test of the model were 13.939 and 0.083, respectively. Figure 3 showed the individual weight of predictors in the model according to the value of R square (Fig. 3-B).

## Discussion

We proposed predictive models in this study to forecast failure, defined as gestational latency of less than 2 days, and success, defined as delivery after 28 weeks of gestation. The models demonstrated excellent and good diagnostic accuracy, respectively. Interestingly, none of the parameters were involved in both early and late outcomes simultaneously. The pathogenesis of delivery immediately following cerclage surgery and that occurring after a certain period of time are believed to differ. There have been several studies on the outcomes associated with rescue cerclage. Some studies have suggested parameters related to the prognosis of rescue cerclage 9, 16, while others have suggested a scoring system to predict prognosis based on physical examination findings 13, and the staging system to assess prognosis in physical examination findings and ultrasound finding 14. However, those studies had only a small number of patients and assessed a limited number of parameters. In contrast, the present study had a larger sample size and used a variety of parameters for analysis.

In this study, the mean gestational age at delivery was 28.3 ± 6.9 weeks of gestation. A retrospective study comparing patients who were managed expectantly and via rescue cerclage revealed that the latter had a mean gestational age at delivery of 30.5-30.6 weeks 8, 17, which was longer than that in our study. In a recent study, after rescue cerclage was established as a treatment, the mean gestational age at delivery was 29.4 weeks 9, similar to our study.

On multivariate analysis, longer gestational age at diagnosis was associated with improved late outcome in this study. It was found to be a critical predictor of success. Previous studies have also reported older gestational age at diagnosis and rescue cerclage were associated with later age at delivery 9, 16. Although some studies have associated nulliparity with poor prognosis 16, this finding remains controversial 9, 14, 16. Our results showed that nulliparity was not related to prognosis for rescue cerclage.

With respect to physical examination, advanced dilatation of cervix was associated with poor prognosis in this study, which was supported by previously published data 9, 11, 12, 14, 18. Poor prognosis was seen in cases wherein the membrane bulged out of the cervix. Compared to previous literatures that relied only on physical examination, the present study also measured the size of the prolapsed membrane and length of functional cervix through ultrasound. We found that the length (B) of the prolapsed membrane and the functional cervical length (C) were all related to the prognosis of rescue cerclage. Less prolapsed membrane and longer functional cervix length was both associated with improved prognosis. Functional cervical length (C) was found to be critical predictor of the late success. These findings suggest that ultrasound examination can be used as an additional tool for prognostication on top of the physical examination. According to the results of multivariate analysis, CRP was useful for predicting failure. There has been research indicating that white blood cell (WBC) count and C-reactive protein (CRP) levels are associated with the prognosis of rescue cerclage 12. Inflammation or infection can be considered as being potentially related to early outcome. A negative result of AmniSure test was related to success in the present study. Despite intact membranes, positive AmniSure test was related to poor outcome. Similarly, another study found that a positive AmniSure test was associated with an increased risk of preterm birth in patients with intact membranes 19. Even in the absence of clinically apparent gross amniotic fluid leakage, a positive AmniSure test would indicate compromised membranes and abnormal fetal membrane system status 20. Therefore, we recommend conducting an AmniSure test before cerclage even if there is no evidence of membrane rupture. In our research, after cerclage, 96% had tocolytics and 98% had antibiotics. For this reason, the effect of such post-cerclage treatments was not analyzed. Studies have shown that these treatments do not affect prognosis 9,12.

In the multivariate analysis, operating time was identified as a prognostic factor for failure in this study. Longer operating time was related to failure, likely because there is more manipulation of the membrane or cervix and longer exposure of the membrane to the air. Therefore, we suggest that the operator must simplify the operation procedure and shorten the operating time as much as possible. The post-cerclage cervical length and height were not related to prognosis in this study. Higher cerclage height was associated with success on univariate analysis, but it was not statistically significant on multivariate analysis. However, a previous study showed that cerclage height was related to prognosis 21. It is thus necessary to conduct further prospective studies with a large sample size.

This study has several limitations. First, since this was a retrospective study with missing data. Second, there were limitations in confirming chorioamnionitis because amniocentesis was rarely performed. Finally, we did not perform an external validation of the suggested models. However, we analyzed and calculated the model using a large, multicenter sample size. We would like to advance our research in the direction of developing a clincal decision support tool for patients with cervical insufficiency, such as the QUantitative Innovation in Predicting Preterm birth” (QUIPP) app, which predicts premature birth in the future 22.

## Conclusion

This study has developed innovative predictive models that can be utilized to objectively predict patient outcomes following rescue cerclage. These models have the potential to be valuable in counseling patients and their families both before and after the procedure, as they can aid in forecasting both early and late outcomes. Specifically, the ability to predict long-term outcomes is expected to greatly assist in improving maternal treatment and subsequent neonatal prognosis. Furthermore, this study reinforces the importance of ultrasound examination and the AmniSure test as necessary components prior to performing rescue cerclage.

## Figures and Tables

**Table 1:** Characteristics of the study population and features of participants experiencing immediate failure (gestational latency < 2 days) following rescue cerclage

Characteristics		All	Immediate failure		
		(N = 98)	Yes (N = 15)	No (N = 83)	*p* value
Age (year)		32.7 ± 4.0	31.6 ± 3.6	32.9 ± 4.1	0.241
BMI (kg/m^2^)	Pre-pregnancy	23.5 ± 4.1	24.0 ± 3.3	23.4 ± 4.2	0.405
Nulliparity	(N, %)	52 (53.1)	11 (73.3)	41 (49.4)	0.102
Gestational age (week)	at diagnosis	21.8 ± 2.6	20.5 ± 3.1	22.0 ± 2.5	0.023^*^
Gestational age (week)	at delivery	28.3 ± 6.9	21.0 ± 3.3	29.7 ± 6.6	<0.001^*^
Symptom at diagnosis	(N, %)	50 (51.0)	13 (86.7)	37 (44.6)	0.008^*^
WBC (1,000 cells/ml)		10.779 ± 2.871	10.697 ± 2.655	10.793 ± 2.653	0.930
CRP (mg/dl)		0.89 ± 1.22	1.46 ± 1.84	0.79 ± 1.06	0.186
Bacterial vaginosis	Positive (N, %)	25 (25.5)	5 (33.3)	20 (24.1)	0.646
Amnisure test	Positive (N, %)	24 (24.5)	8 (53.3)	16 (19.3)	0.037^*^
Physical examination	prolapsed membrane (N, %)	88 (89.8)	14 (93.3)	74 (89.2)	0.438
	Cervical dilatation (cm)	2.85 ± 1.21	3.46 ± 1.18	2.75 ± 1.19	0.049^*^
	Cervical dilatation >2cm (N, %)	75 (72.8)	11 (73.3)	63 (71.6)	0.536
Ultrasound finding (cm)	A	2.27 ± 1.82	3.96 ± 2.02	1.98 ± 1.63	<0.001^*^
At diagnosis	B	1.32 ± 1.43	2.95 ± 2.04	1.04 ± 1.08	0.003^*^
	C	3.67 ± 1.05	3.20 ± 0.74	3.75 ± 1.08	0.040^*^
Post-cerclage	Cervical length	2.15 ± 0.93	2.63 ± 1.44	2.10 ± 0.87	0.346
ultrasound finding (cm)	Cerclage height	1.36 ± 0.50	1.14 ± 0.62	1.38 ± 0.49	0.195
Operation time^1^ (min)		27.7 ± 18.6	40.0 ± 22.1	25.6 ± 17.2	0.005^*^

Data was described as mean ± standard deviation (SD), and N (proportion). A, width of prolapsed membrane of ultrasound; B, length of prolapsed membrane of ultrasound; C, length of functional cervix of ultrasound; BMI, body mass index; GA, gestational age; WBC, white blood cell count; CRP, C-Reactive protein. ^*^p value < 0.05; ^†^Data was unavailable in 2 patients.

**Table 2:** Characteristics of the study population with a satisfactory latency period (≥8 Weeks) and long-term success (delivery ≥ 28 weeks of gestation) following rescue cerclage.

Characteristics		Latency period				Gestational age at delivery		
		< 8weeks (N = 61)	≥8weeks (N = 37)	*p* value		<28weeks (N = 53)	≥28weeks (N = 43)	*p* value
Age (year)		33.5 ± 4.7	31.9 ± 2.7	0.049^*^		33.7 ± 4.4	31.9 ± 3.3	0.028^*^
BMI (kg/m^2^)	Pre-pregnancy	24.0 ± 4.0	22.5 ± 3.6	0.088		23.6 ± 3.5	22.7 ± 4.0	0.296
Nulliparity	(N, %)	36 (59.0)	16 (43.2)	0.216		25 (47.1)	22 (51.1)	0.821
Gestational age (week)	at diagnosis	21.4 ± 2.6	22.4 ± 2.6	0.084		21.0 ± 2.6	22.9 ± 2.2	<0.001^*^
Symptom at diagnosis	(N, %)	53 (86.9)	17 (45.9)	0.008^*^		34 (64.2)	14 (32.6)	0.004^*^
WBC (1,000 cells/ml)		11.175 ± 3.207	9.877 ± 0.248	0.045^*^		11.138 ± 3.044	10.133 ± 2.688	0.047^*^
CRP (mg/dl)		1.13 ± 1.59	0.56 ± 0.53	0.021^*^		1.13 ± 1.57	0.57 ± 0.51	0.008^*^
Bacterial vaginosis	Positive (N, %)	16 (26.2)	8 (21.6)	0.813		15 (28.3)	9 (20.9)	0.499
Amnisure test	Positive (N, %)	30 (49.2)	11 (29.7)	0.458		17 (32.0)	5 (11.6)	0.002^*^
Physical examination	Prolapsed membrane (N, %)	58 (95.1)	30 (81.1)	0.083		48 (90.5)	36 (83.7)	0.487
	Cervical dilatation (cm)	3.00 ± 1.22	2.48 ± 1.17	0.067		3.13 ± 1.24	2.59 ± 1.13	0.041^*^
	Cervical dilatation >2cm (N, %)	53 (86.9)	26 (70.3)	0.178		40 (75.4)	30 (69.7)	0.356
Ultrasound finding (cm)	A	2.74 ± 1.87	1.44 ± 1.62	0.003^*^		2.83 ± 1.87	1.57 ± 1.57	0.001^*^
at diagnosis	B C	1.80 ± 1.65 3.61 ± 1.03	0.67 ± 0.99 3.69 ± 1.15	0.001^*^ 0.761		1.82 ± 1.61 3.78 ± 1.01	0.80 ± 1.05 3.57 ± 1.15	0.001^*^ 0.379
Post-cerclage	Cervical length	2.06 ± 0.96	2.33 ± 0.82	0.209		2.05 ± 1.04	2.25 ± 0.85	0.334
ultrasound finding (cm)	Cerclage height	1.25 ± 0.46	1.57 ± 0.42	0.005^*^		1.18 ± 0.53	1.51 ± 0.44	0.005^*^
Operation time^1^ (min)		25.2 ± 12.1	21.0 ± 16.6	0.213		31.7 ± 20.3	22 ± 15.5	0.012^*^

Data was described as mean ± standard deviation (SD), and N (proportion). A, width of prolapsed membrane of ultrasound; B, length of prolapsed membrane of ultrasound; C, length of functional cervix of ultrasound; BMI, body mass index; GA, gestational age; WBC, white blood cell count; CRP, C-Reactive protein. ^*^p value < 0.05; ^†^Data was unavailable in 2 patients.

**Table 3:** Multivariate binary logistic regression analysis for risk factors to predicting immediate failure (gestational latency < 2days) and long-term success (delivery ≥ 28 weeks of gestation) following rescue cerclage.

		Immediate failure								Long-term success					
		B	OR	95% CI	*p* value					B			OR	95% CI	*p* value
CRP (mg/dl)		0.579	1.784	1.029 - 3.096	0.031										
B (cm)		1.009	2.740	1.468 - 5.128	< 0.001										
Operation time (min)		0.049	1.060	0.991 - 1.112	0.051										
Gestational age at diagnosis (week)												0.465	1.592	1.200 - 2.112	< 0.001
Cervical dilatation (cm)												- 0.784	0.457	0.230 - 0.908	0.025
C (cm)												0.638	1.892	1.147 - 3.123	0.003
Amnisure test (negative)												0.162	1.176	0.991 - 1.395	0.063
															

Adjusted by maternal age, BMI (body mass index), cervical length before rescue cerclage, and parity. B, length of prolapsed membrane of ultrasound; C, length of functional cervix of ultrasound; CRP, C-Reactive protein; CI, confidence interval; OR, odds ratio.

**Table 4:** AUROC analysis of predictive models for immediate failure (gestational latency < 2 days) and long-term success (delivery ≥ 28 weeks of gestation) after rescue cerclage.

Predictive model		Cut off	Threshold	Sensitivity	Specificity	PPV	NPV	Diagnostic accuracy	95% CI
Immediate failure	CRP (mg/dl)	0.056	>4.3	100.0	70.6	31.0	100.0	0.912	0.834-0.989
	B (cm)		>2.9						
	Operation time (min)		>20						
Long-term success	Gestational age at diagnosis (week)	0.439	>22.3	88.4	73.3	82.6	81.5	0.872	0.788-0.956
	Cervical dilatation (cm)		< 3						
	C (cm)		>4						
	Amisure test		Negative						

B, length of prolapsed membrane of ultrasound; C, length of functional cervix of ultrasound; CI, confidence interval; CRP, C-Reactive protein; PPV, positive predictive value; NPV, negative predictive value
